# The catalytic activity and secretion of zebrafish RNases are essential for their *in vivo* function in motor neurons and vasculature

**DOI:** 10.1038/s41598-018-37140-2

**Published:** 2019-02-01

**Authors:** Ross Ferguson, Daniel E. Holloway, Anand Chandrasekhar, K. Ravi Acharya, Vasanta Subramanian

**Affiliations:** 1 Department of Biology and Biochemistry, University of Bath, Bath, BA2 7AY UK; 2 Division of Biological Sciences and Bond Life Sciences Center, University of Missouri, Columbia, MO 65211-7310 USA

## Abstract

Angiogenin (*hANG*), a member of the Ribonuclease A superfamily has angiogenic, neurotrophic and neuroprotective activities. Mutations in hANG have been found in patients with Amyotrophic lateral sclerosis (ALS). The zebrafish (*Danio rerio*) *rnasel-1*, *2* and 3 are orthologues of *hANG* and of these only Rnasel-1 and Rnasel-2 have been shown to be angiogenic. Herein we show that NCI-65828, a potent and specific small molecule inhibitor of hANG inhibits Rnasel-1 to a similar extent. Treatment of early zebrafish embryos with NCI-65828, or with terrein, a fungal metabolite which prevents the secretion of hANG, resulted in spinal neuron aberrations as well defects in trunk vasculature. Our detailed expression analysis and inhibitor studies suggest that Rnasel-1 plays important roles in neuronal migration and pathfinding as well as in angiogenesis in zebrafish. Our studies suggest the usefulness of the zebrafish as a model to dissect the molecular consequences of the ANG ALS variants.

## Introduction

**Figure 1:**
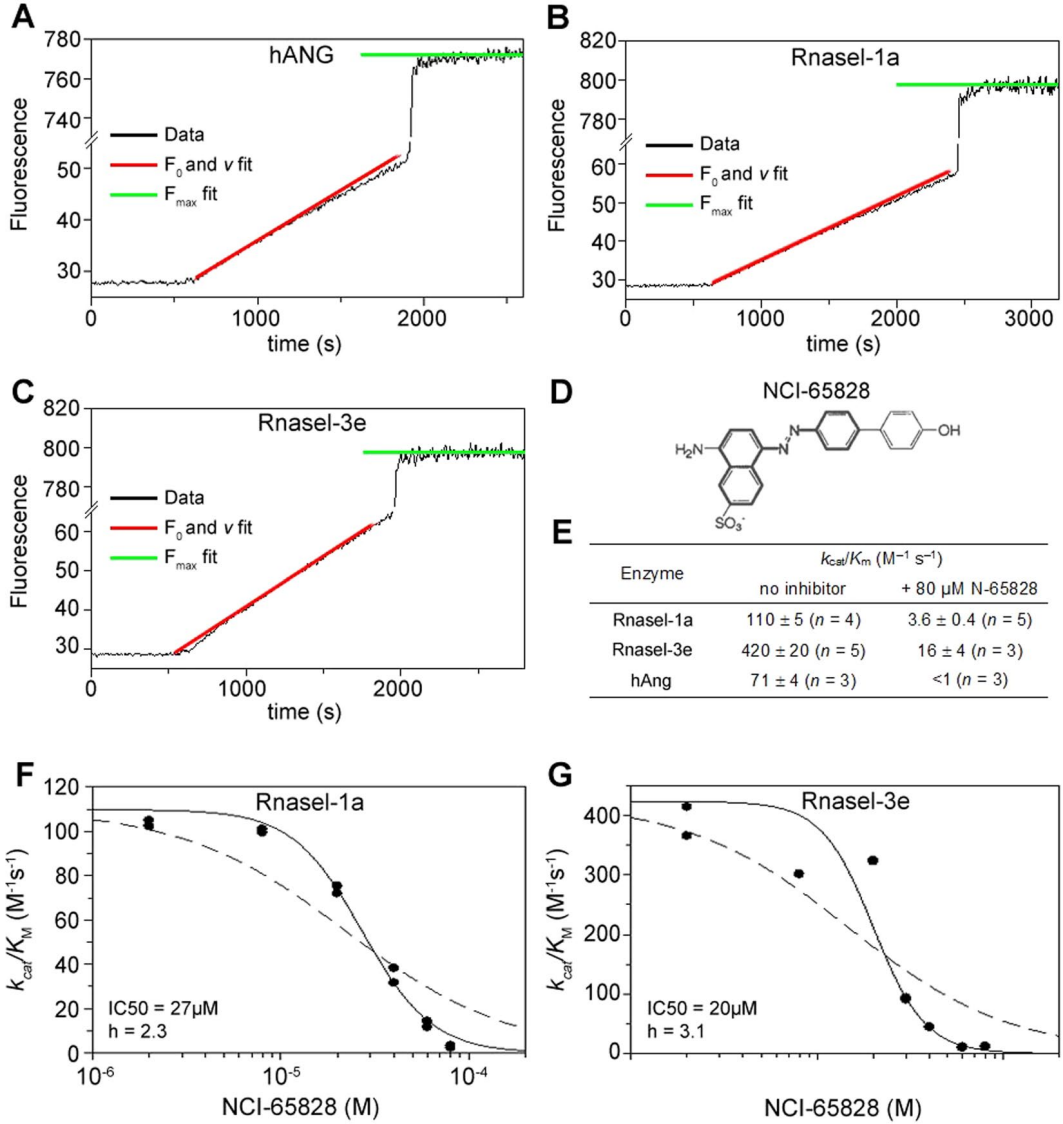
Inhibition of Rnasel-1 and -3 by the small molecule inhibitor NCI-65828. (**A**–**C**) Fluorescence-based RNase activity assays shows Rnasel-1a and -3e have similar F_0_ and v fit slopes indicating similar activities. (**D**) Chemical structure of NCI-65828, a specific inhibitor of human ANG which also inhibits the ribonuclease activity of Rnasel-1a and -3e at 80 µM. (**E**) Enzyme kinetic data for Rnasel and hANG native protein and in the presence of NCI-65828. (**F**,**G**). Dose response plots for Rnasel-1a and -3e. IC_50_ value determined for both proteins.

Human angiogenin (hANG), a potent angiogenic factor is a single chain polypeptide of Mr ~ 14400 first isolated from human colon adenocarcinoma cell line (HT-29) conditioned media^1,2^. It is a member of the Ribonuclease A (RNase A, also known as RNase 5) superfamily and has weak ribonucleolytic (catalytic) activity^3^. Angiogenin is a secreted protein also found in human plasma^4^ and is produced as a pre-protein with a signal sequence^5^. Secreted hANG is taken up by cells in culture and translocated to the nucleus^3,6,7^. The catalytic activity and nuclear translocation are both essential for its angiogenic activity^8^. Like RNase A, ANG cleaves preferentially on the 3′ side of pyrimidine and follows a transphosphorylation/hydrolysis mechanism^3^. An extensive high-throughput screening assay (18,310 compounds from the National Cancer Institute (NCI, USA) Diversity Set and ChemBridge DIVERSet) has identified NCI-65828 (8-amino-5-(4′-hydroxybiphenyl-4-azo) napathlene-2-sulphate) **(**Fig. 1D**)** as a selective and potent cell permeable inhibitor of the catalytic activity (*K*_i_ = 81 µM) of hANG and targets the active site^9^. In another study, the fungal metabolite and antibiotic, terrein (4,5-dihydroxy-3-[(E)-prop-1-enyl]cyclopent-2-en-1-one) **(**Fig. 3E**)** has been shown to specifically inhibit the secretion of ANG^10^.

Angiogenin has also been shown to be a neurotrophic and neuroprotective factor^11–13^. We and others have shown that hANG is expressed in the developing nervous system and in neurons^11,14,15^. We have also shown previously that NCI-65828 inhibits neurite extension in cell cultures but not differentiation to neurons^11^. Furthermore, Wei *et al*.^16^ have shown that hANG plays a role in cell migration through its interaction with β-actin and α-actinin at the leading edge of migrating cells and cell migration is compromised in hANG deficient cells.

hANG is involved in many disease conditions and has been shown to be upregulated in various human cancers, diabetic retinopathy and arthritis^17^. hANG has also been implicated in neurodegenerative diseases as mutations in hANG have been identified in patients with Amyotrophic Lateral Sclerosis (ALS), Parkinson’s disease and Fronto-Temporal Dementia (FTD)^15,18–22^. Mutations in *hANG* found in familial and sporadic ALS patients affect the active site, the signal sequence, important functional residues as well as the nuclear localization signal (NLS)^15,18–23^. In addition, in an extensive study of selected ANG-ALS variants we correlated the effects of the structural changes on neuronal survival and the ability to induce stress granules in neuronal cell lines. We also established that ANG-ALS variants that affect the structure of the catalytic site which either decrease or increase in the RNase activity affect neuronal survival. Neuronal cell lines expressing the ANG-ALS variants also lacked the ability to form stress granules^24^.

Zebrafish (*Danio rerio****)*** RNase-like proteins (Rnasel1, 2 and 3) are hANG like RNases identified in zebrafish^25–28^. The Rnasels are secreted RNases and have a signal sequence, the “CKXXNTF” signature motif and the catalytic triad, as well as six conserved cysteine residues similar to hANG (RNase 5)^29^. Previously, we have shown (based on a detailed structure-function study) that Rnasel-1a cleaves tRNA with a specific activity similar to hANG. This is consistent with the finding that the active site in Rnasel-1a is blocked by its C-terminus as in hANG^27^. This is in contrast to Rnasel-3e in which the active site is open and which has 17–20 fold more RNase activity towards tRNA^27^.

In this study, we have used the compound NCI-65828, a small molecule that inhibits the enzymatic activity and terrein, a fungal metabolite known to prevent the secretion of hANG by prostate cancer cell lines, to explore if both the enzymatic activity and the secretion of hANG are essential for its *in vivo* neuronal and angiogenic functions. For our model systems, we used neuronal cell lines stably expressing HA epitope tagged mouse Ang1 (mAng1) and *Tg(fli1a:EGFP)* zebrafish which express EGFP in the vascular system^30^. The nervous system of the zebrafish is well characterised, and its relatively simple neuromuscular organization makes it an ideal model to study neurodegenerative disorders^31,32^. Prior to carrying out a detailed functional study, we first investigated whether NCI-65828 inhibits zebrafish RNases.

Here we report that NCI-65828 inhibits Rnasels and that human neuronal cells exposed to terrein accumulate mAng1. We also show that *in vivo* inhibition of the RNase activity of Rnasels and their secretion leads to defective development of spinal motor axons and intersegmental vessels. Our results show that both the catalytic activity and the secretion of ANG-like Rnasels play important roles during development of the zebrafish nervous system and vasculature.

## Results

### NCI-65828 is a potent inhibitor of the ribonucleolytic activity of Rnasels

Prior to studying the effects of NCI-65828 (8-amino-5-(4′-hydroxybiphenyl-4-azo) napathlene-2-sulphate) (Fig. 1D), a selective and potent cell permeable inhibitor of the catalytic activity of hANG, on zebrafish motor neurons and vasculature we sought to establish if indeed NCI-65828 is also able to inhibit the enzymatic activity of zebrafish Rnasels.

We initially established the time courses for cleavage of 6-FAM–mAmArCmAmA–Dabcyl (a fluorogenic substrate) by Rnasels -1a, -3e and hANG which enabled clear measurements of *v*, *F*_0_, and *F*_max_ (Fig. 1A–C). These curves provided highly reproducible estimates of *k*_cat_/*K*_m_ (Fig. 1F). Using this substrate (which has its cleavable bond between a cytidine and an adenine residue, and is thus tailored for hANG), the specific activities of Rnasel-1a and hANG were very similar. This is also the case for the cleavage of tRNA by these two enzymes^27^. This differs slightly from the result obtained with 6-FAM–dArUdAdA–6-TAMRA (cleavable between uridine and adenine), which is cleaved ~3-fold more readily by Rnasel-1a^25^, although this is consistent with structural features in the B_1_ subsite of Rnasel-1a that may promote uridine binding^27^. The specific activity of Rnasel-3e with the present substrate is 4- and 6-fold greater than that of Rnasel-1a or hANG, respectively. This is fairly similar to the profile obtained with 6-FAM–dArUdAdA–6-TAMRA^25^ but is somewhat less marked than that obtained with tRNA, which is cleaved 17–20-fold more readily by Rnasel-3e^27^.

Having established the time course of RNase activity, we studied the effect of 80 µM NCI-65828 (Fig. 1D), previously reported to inhibit hANG, on the ribonucleolytic activity of Rnasels^9^. 6-FAM fluorescence was quenched significantly by 80 µM NCI-65828. Assay sensitivity could be restored satisfactorily by increasing the fluorimeter’s emission slit width, and all data were validated by verifying that the cleavage-induced increase in fluorescence (*i*.*e*. the value of *F*_max_/*F*_0_) fell in the normal range (25–30). In the presence of 80 µM NCI-65828, the ribonucleolytic activities of Rnasel-1a and -3e were only 3–4% of normal, indicating substantial inhibition (Fig. 1E).

Dose-response plots were constructed to determine the strength and mode of inhibition experienced by Rnasels-1a and -3e in the presence of NCI-65828 (Fig. 1F,G). A sigmoid curve of standard slope, *i*.*e*. *h* = 1 (dashed lines) was obtained. However, a better fit is obtained by permitting a steeper slope, giving estimates of IC_50_ = 27 µM (*h* = 2.3) for Rnasel-1a, and IC_50_ = 20 µM (*h* = 3.1) for Rnasel-3e (solid lines).

### Spatio-temporal expression pattern of zebrafish *rnasel1–3*

**Figure 2:**
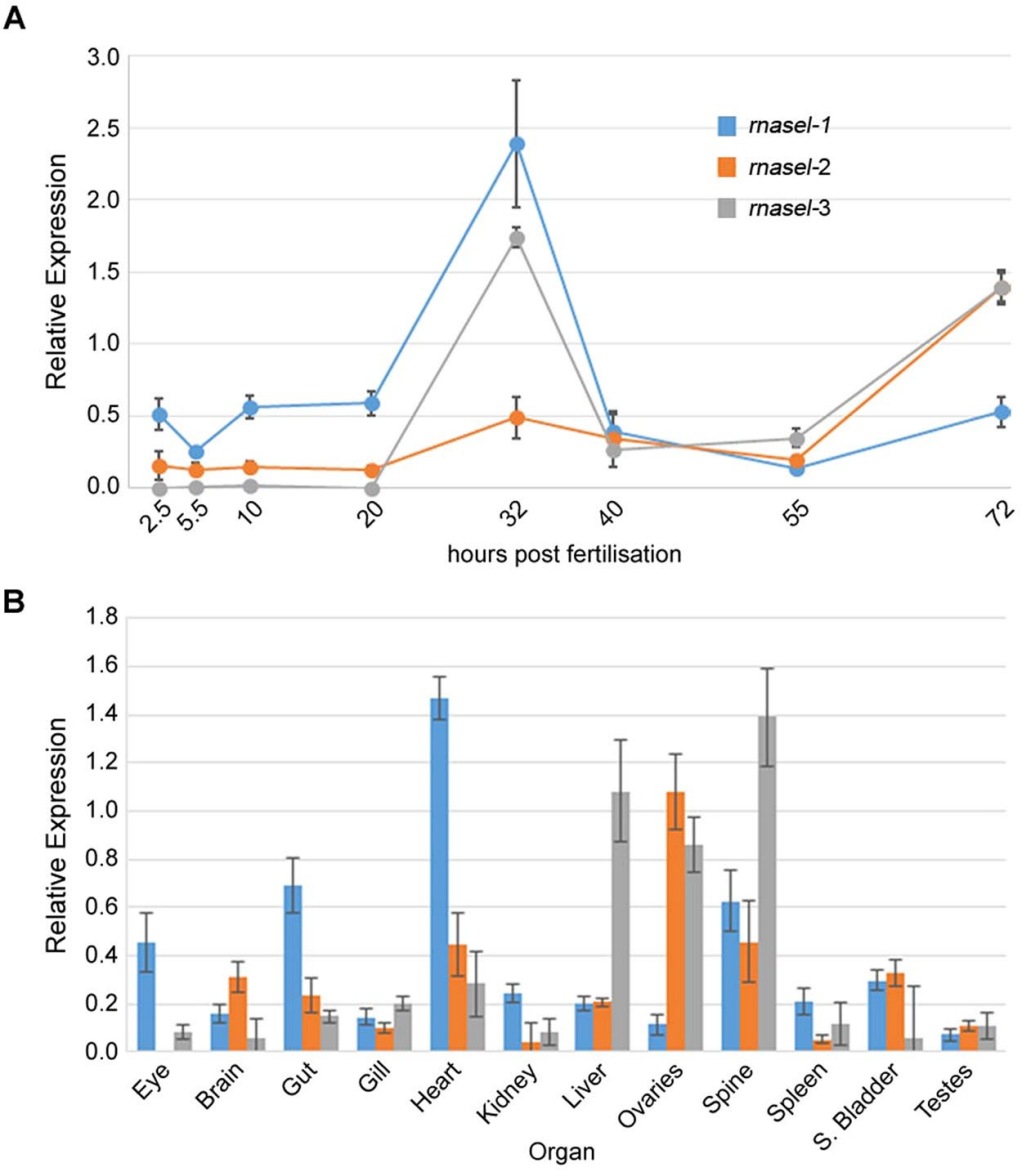
Expression of *rnasel1–3* in the developing and adult zebrafish. (**A**) qRT-PCR for *rnasel* transcripts corresponding to specific isoforms during development shows a peak in *rnasel* 1 and 3 expression around 32 hpf and a second peak of *rnasel* 2 and 3 at 72 hpf. (**B**) qRT-PCR in adult zebrafish organs shows strong expression of *rnasel* 1 and 3 in the spine. All PCRs in triplicate from two independent pools of material. Expression relative to 18 s rRNA transcript. Error bars SEM.

We carried out a systematic analysis of *rnasel* expression at various embryonic stages as well as in adult organs as described in materials and methods (see online methods) which would enable us to ascertain which of the *rnasel*s was likely to be the cause of the *in vivo* defects when inhibited by NCI-65828. Expression of all three *rnasel*s was low at 2.5 hours post fertilization (hpf) - the earliest embryonic stage analysed, though *rnasel*-1 was higher than -2 and -3. Expression of *rnasel*-1 and -3 dramatically increased after 20 hpf, with a peak at 32 hpf after which levels of both *rnasel*-1 and -3 dropped to initial levels. A small increase in *rnasel*-2 levels occurred at 32 hpf and peaked at 72 hpf. This peak of expression at 72 hpf was also seen for *rnasel*-3. The fall in expression levels seen between 32 and 40 hpf for *rnasel*-1 expression was maintained at consistent levels until 55 hpf after which a small increase was seen, mirroring that of *rnasel*-2 and -3 (Fig. 2A).

The expression levels of *rnasel*s relative to each other observed at 72 hpf are similar to the overall pattern seen in adult fish where increased expression of *rnasel*-2 and -3 is seen over *rnasel*-1 (Fig. 2B). *rnasel*-2 and -3 both appear to be strongly expressed in the ovaries with very little expression of *rnasel*-1. Conversely little expression of any *rnasel* is seen in the testes. *rnasel*-1 appears to be strongly expressed in the eye, gut, heart and spinal chord in the adult fish and seems to be the dominant transcript in the eyes, gut and heart. The highest overall expression of all three isoforms appears to be in the spinal cord, while the highest expression of the individual isoforms is seen in the heart for *rnasel*-1, ovaries for *rnasel*-2 and liver for *rnasel*-3. Low expression with comparable levels between transcripts is seen in organs such as the gills, spleen and swim bladder (Fig. 2B).

### Terrein treatment of SH-SY5Y mAng1HA cells leads to accumulation of intra-cellular Ang

**Figure 3:**
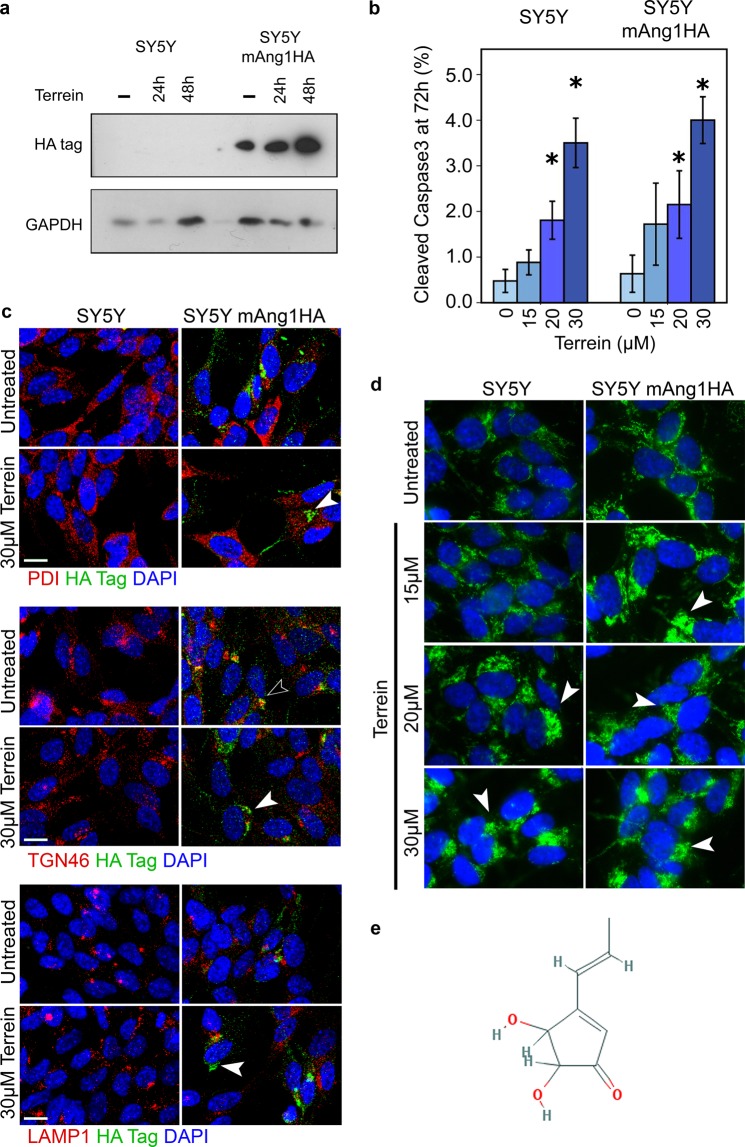
Effect of terrein on the secretion of ANG by SH-SY5Y mAng1HA cells. (**A**) Western blot of SH-SY5Y and SH-SY5Y expressing HA-tagged ANG (mAng1HA) treated with 30 µM terrein for 24 or 48 hours shows increased retention of ANG compared to untreated (−). GAPDH used as loading control. (**B**) Terrein treatment lead to an increase in apoptosis correlating with increasing concentration, as shown by quantification of cells with DAPI stained nuclei positive for cleaved caspase 3 after treatment for 72 h (Mean results from four fields in two experiments containing an average of 640 cells/field). Error bars: SEM, ∗P < 0.05. (**C**) Immunostaining for HA tagged mAng1 shows increased intracellular accumulation upon terrein treatment (white arrows). Co-staining with organelle markers for the ER (PDI), Golgi (TGN46) or Lysosome (LAMP1) shows co-localisation with TGN46 which is lost upon terrein treatment (black arrow). (**D**) Terrein treatment also results in a redistribution of apoptosis-inducing factor (AIF) to a peri-nuclear position, but translocation into the nucleus is inhibited. This effect increases with terrein concentration and occurs at lower concentrations in mAng1-overexpressing cells (white arrows). (**E**) Chemical structure of terrein.

Prior to investigating the effects of terrein on neuronal development and vascularization in zebrafish, we first investigated if indeed treatment with terrein affected the secretion of Ang using a SH-SY5Y cell line constitutively expressing a HA epitope tagged mouse mAng1 which we will refer to as SH-SY5Y mAng1HA. We analysed the intra-cellular accumulation of the HA tagged mAng1 in these cells by Western blotting. Intracellular levels of mAng1HA increased over the course of the 24 or 48 h treatment with 30 μM terrein relative to GAPDH loading (Fig. 3A).

The adverse effects, if any, due to accumulation of Ang in the SH-SY5Y mAng1HA cells treated with terrein was investigated. There were no obvious morphological changes or increased incidence of cleaved caspase 3 positive cells in both untreated cells and cells cultured in terrein for 24 h (Fig. 3B). However, with a longer terrein treatment of 72 h, we observed an increase in cleaved caspase 3 positive cells as compared to the control. The SH-SY5Y mAng1HA cell line had significantly more cleaved caspase 3 positive cells than untreated SH-SY5Y mAng1HA cells at both 20 µM and 30 µM of terrein compared by ANOVA (P < 0.05). The most significant effect was observed at the highest concentration (30 µM) (Fig. 3B).

The intracellular distribution of HA tagged mAng1 in the terrein treated SH-SY5Y mAng1HA cells was different when compared with the untreated cells. There was a high incidence of large aggregates in the cell bodies, which increased in number at higher terrein concentrations. Co-staining with organelle markers showed that a significant amount of mAng1HA no longer co-localised with TGN46. No co-localisation seen with either PDI or LAMP1 (Fig. 3C).

It has previously been reported that in P19 embryonal carcinoma cells exposed to high levels of ANG, the apoptosis inducing factor (AIF) does not translocate to the nucleus^33^. In control SH-SY5Y mAng1HA cells where secretion of ANG is not inhibited, AIF is seen evenly distributed throughout the cell body. However, in terrein treated SH-SY5Y mAng1HA cells with an accumulation of mAng1HA, AIF aggregates are localised adjacent to the nucleus (Fig. 3D).

### Blocking the RNase activity and secretion of Rnasels in zebrafish embryos leads to motor neuron and vascular defects

**Figure 4:**
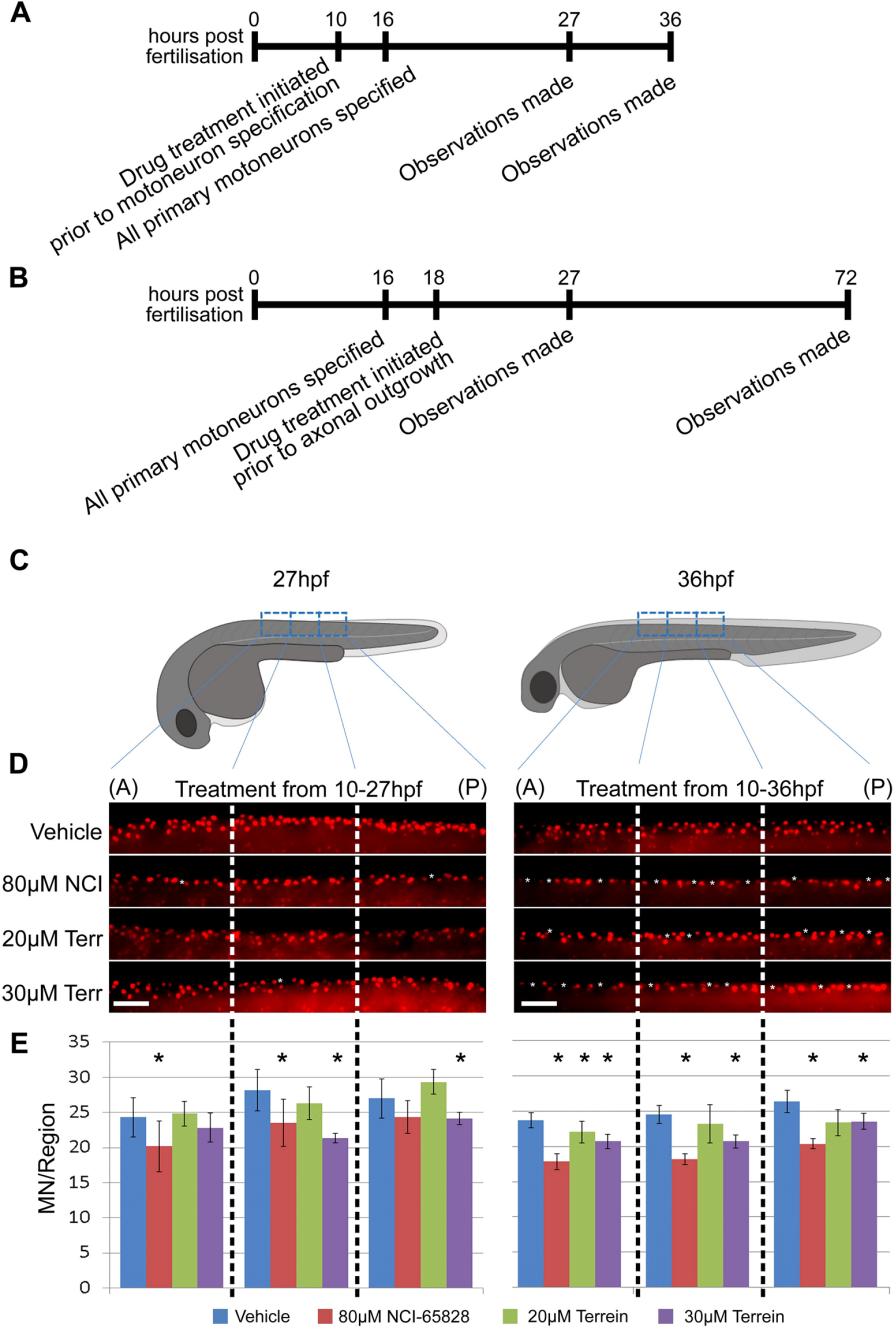
Effects of NCI-65828 and terrein on zebrafish spinal neurons. (**A**) Experimental plan for drug treatment of embryos at 10 hpf prior to specification of primary motor neurons and primary motor axon outgrowth. (**B**) Experimental plan for drug treatment of embryos at 18 hpf after specification of primary motor neurons but prior to primary motor axon outgrowth. (**C**) Diagrams of embryos at 27 and 36hpf with regions analysed highlighted (**D**) Islet1 immunostaining at 27 and 36 hpf in the developing spinal cord of embryos treated from 10 hpf with NCI-65828 or terrein. Increased gaps between islet^+^ nuclei seen (white asterisks). A/P, anterior/posterior. (**E**) Quantification of motor and Rohon-Beard sensory neurons in the spinal cord at the indicated time-points over the three regions anterior (A) to posterior (P) from 20 embryos (two replicates with 10 embryos each) shows a significant decrease (*p < 0.05) after treatment at 10 hpf. Regions counted and binned as shown by dashed regions in panel C and D.

Transgenic *Tg(fli1a:EGFP)*^30^ embryos were used to assess both vascular and motor neuron development following treatment with NCI-65828 or terrein. We treated zebrafish embryos with NCI-65828 or terrein at two critical stages in the formation of motor neurons - at 10 hpf when primary motor neurons (1°MNs) are generated in the nascent brain and spinal cord, and at 18 hpf when they extend axons^34,35^ (Fig. 4A,B).

The dorsal aorta also puts forth sprouts at around 20 hpf (after 1° MN axon extension) by angiogenesis. These sprouts lengthen longitudinally to connect up and form the dorsal longitudinal anastomotic vessels^36–38^.

### Effects on motor neurons on treatment with NCI-65828 or terrein prior to their specification

*Tg(fli1a:EGFP)* zebrafish embryos were treated either with NCI-65828 or terrein at 10 hpf and allowed to develop to 27 and 36 hpf stages (Fig. 4A,B). We focused on a subset of primary motor neurons. Cell bodies of the caudal primary neurons (CaP) and the variable primary neurons (VaP) located in the middle of each of the spinal cord hemisegment express the LIM homeodomain protein islet2^39^. Control and inhibitor treated embryos were stained with mouse αIslet antibody, labelling both Rohon-Beard sensory neurons in the dorsal spinal cord and motor neurons prior to their migration to the ventral horn. We counted the number of islet^+^ nuclei in the trunk (Fig. 4C) and found a significant reduction in the number of islet^+^ nuclei in embryos treated with both NCI-65828 and 30 µM terrein (Fig. 4D,E**)** at 36hpf. We also observed misplaced and missing islet^+^ cells.

**Figure 5:**
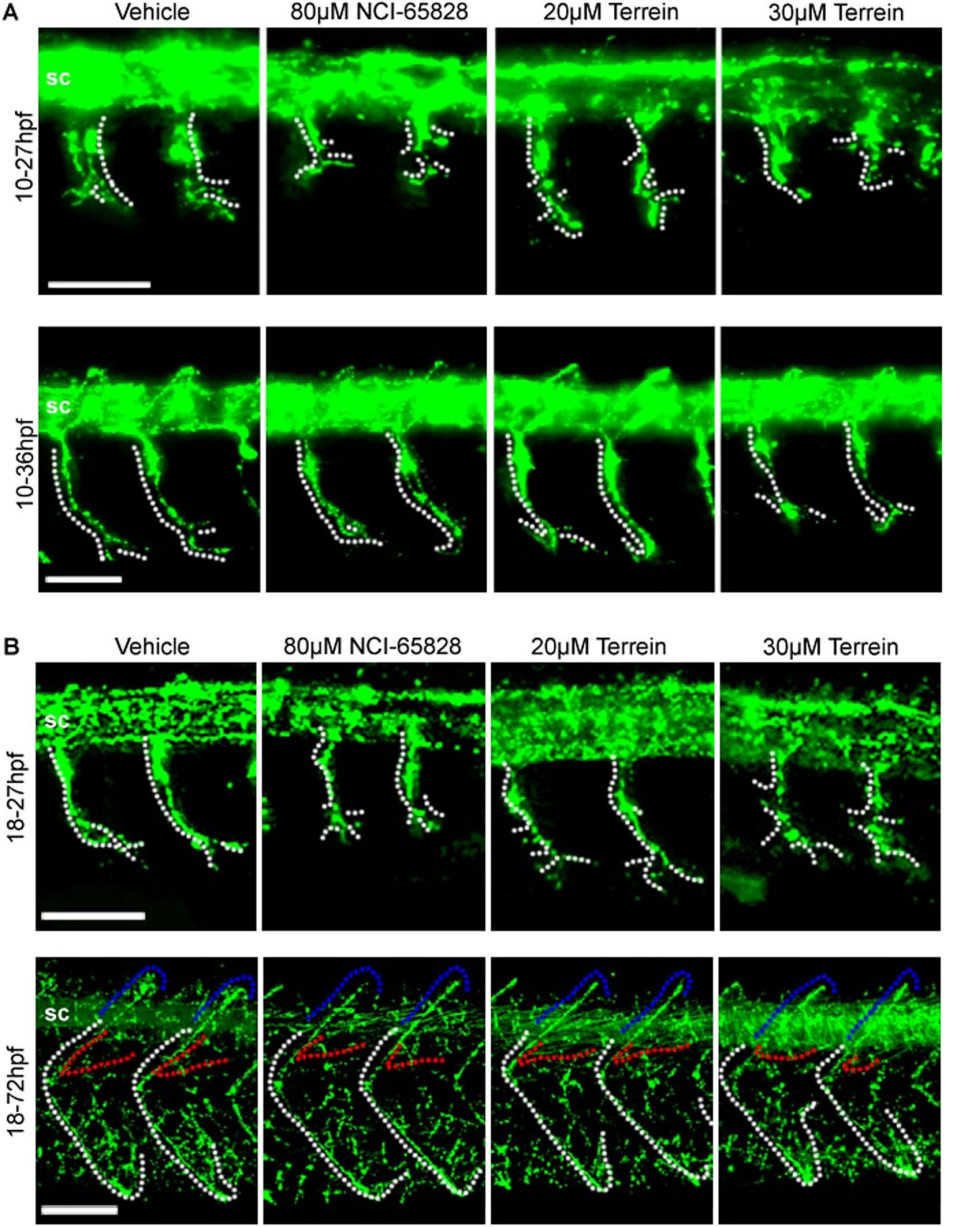
Axonal defects in zebrafish embryos treated with NCI-65828 and terrein. Immunostaining for Znp1 in Fli:GFP zebrafish treated with NCI-65828 or terrein from (**A**) 10 hpf or (**B**) 18 hpf. Znp1 staining shows defects in the extension and branching of the caudal primary motor neuron (CaP, white dotted lines) extending from the spinal cord (sc). In addition to shortened axons, additional aberrant branching can be seen in treated embryos at positions more proximal to the spinal cord when compared to untreated, which branch only at the distal regions of the axon. Axons in embryos treated at 10 hpf also appear to loop and branch anteriorly at 36 hpf frequently while untreated continue to extend towards the posterior. Treatment from 18 hpf still results in reduced axon length at 27 hpf but to a lesser degree than those treated at 10 hpf. Aberrant branching is still present but with increased branching at the distal tip. By 72 hpf CaP axons appear positioned normally in treated and untreated alike. Observations from 10 embryos in two replicates show significant effects. Detailed quantification can be found in Figure S1. Defects are still seen in the rostral and medial primary motor neurons (RoP and MiP, red and blue dotted lines) and secondary motor neurons (not shown). See Figure S2 for quantification. Scale bars 50 µm.

Inhibitor-treated and control embryos were stained with mouse αZnp1 to visualise CaP motor axons and rabbit αGFP to visualise the vascular system. In embryos treated with inhibitors from 10 hpf, the spinal cord can be seen clearly by Znp1 staining (Fig. 5A). No differences were seen between the longitudinal tracts of the spinal cord in treated and untreated embryos. In both controls and embryos treated with inhibitors, the primary motor neuron axon length correlated with A-P position where anterior axons are more developed (Fig. 5A, dotted lines). Axons in NCI-65828 and terrein treated embryos projected from the spinal cord at the same positions within each somite as untreated and the proximal part of the axon projected at a similar angle dorso-ventrally in all cases. Similarly in all cases each axon had an increasing posterior curve as development continued to 36 hpf, however the distal tip of axons treated with NCI-65828 or terrein were seen to loop back towards the anterior frequently while untreated axon continued extend towards the posterior. At 27 hpf, axons in untreated embryos branched at the distal tip while treated embryos frequently branched in more medial regions of the axons in both anterior and posterior directions. These aberrant medial branches were not noticeable at 36 hpf, where increased branching is seen at the distal tip (Fig. 5A).

Axonal length measured in the trunk of control 27 hpf embryos ranged from 101.4 µm ± 26.6 SD to 85.6 µm ± 23.5 SD along the anterior-posterior axis (Supplementary Fig. S1). In comparison, axonal length at 27hpf embryos treated with NCI-65828 at 10 hpf was significantly decreased ranging in length from a maximum of 68.3 µm ± 14.7 SD at the anterior to 45.8 µm ± 5.1 SD towards the posterior. Similar effects were in embryos incubated in terrein (either 20 µM or 30 µM) from 10hpf (75.4 µm ± 14.2 SD to 43.9 µm ± 18.1 SD and 75.1 ± 14.0 SD to 49.8 µm ± 9.4 SD respectively). By 36hpf, the axonal length of NCI-65828 treated embryos was similar to untreated embryos suggesting that at earlier stages axonal extension was retarded by NCI-65828 treatment but accelerated between 27–36 hpf. However, axons in terrein treated embryos remained shorter when compared with untreated and NCI-65828 treated embryos. Ectopic branching was more frequent in embryos treated with NCI-65828 and 30 µM terrein in posterior axons (Supplementary Fig. S1).

### Effects on motor neurons on treatment with NCI-65828 and terrein after their specification

Zebrafish embryos treated with NCI-65828, 20 µM or 30 µM terrein at 18.5 hpf were allowed to develop to 27 and 72 hpf. Embryos exhibited no gross morphological abnormalities when observed by brightfield microscopy at 27 hpf. Embryos under all conditions at this time point moved spontaneously, had beating hearts and visible dorsal, ventral, yolk sac and head vasculature.

Embryos incubated in inhibitors from 18 hpf, had a normal spinal cord similar to embryos treated at 10hpf and to control embryos. The CaP axon out-growth position or its angle relative to the spinal cord at 27 hpf was also similar to the control (Fig. 5B**)**. The axons were shorter though not as severely affected as in embryos exposed to inhibitors from 10 hpf. Treatment with NCI-65828 or terrein caused branching in incorrect medial positions in both anterior and posterior segments. Increased branching was particularly noticeable in 20 µM terrein treated embryos which may be due to the presence of relatively longer axons when compared to those seen in NCI-65828 or 30 µM terrein treated embryos.

Immunostaining for Znp1 revealed a retardation of axon outgrowth, with lengths of trunk CaP axons decreasing in inhibitor treated embryos when compared with untreated embryos particularly in more posterior segments. The length of CaP axons in untreated embryos was consistent between each segment while axons in NCI-65828 treated embryos were significantly affected. This effect was also seen in embryos treated with 20 and 30 µM terrein. The effect on axonal growth was less severe at 20 µM terrein but more pronounced at 30 µM terrein and the retardation of axon outgrowth was comparable to that in embryos incubated in NCI-65828 (Supplementary Fig. S1). Few branches were observed on axons of untreated control embryos at 27 hpf. Treatment with NCI-65828 resulted in a loss of branches from the more posterior neurons (Supplementary Fig. S1). Unexpectedly, treatment with 20 or 30 µM terrein resulted in increased branching across all axons.

The positioning of caudal, rostral and medial primary motor neuron (Fig. 5B**;** CaP, RoP and MiP axons, white, red and blue dotted lines)^40^ were indistinguishable between treated and untreated embryos by 72 hpf. CaP, RoP and MiP motor neuronal axons in treated and control embryos show the same angle of projection, position of the curve towards the posterior at the midline (in the case of the CaP and RoP), and the curve of the MiP ventrally. Later born secondary motor neuron axons however, were found to be significantly longer in treated embryos under all three conditions with increased branching, which was more pronounced in terrein treated embryos (Supplementary Fig. S2**)**.

Znp1 staining also revealed mean CaP axon lengths to be consistent across trunk region analysed, both in the untreated controls and in embryos treated with inhibitors at 18.5 hpf and observed at 72 hpf. However, the RoP axons were longer after NCI-65828 treatment when compared to controls (249.7 µm ± 46.4 SD) or 20 and 30 µM terrein (Supplementary Fig. S2**)**. No difference in branching of RoP axons was seen with 20 or 30 µM terrein treatment when compared to controls. However, incubation in NCI-65828 caused an increase in branching, particularly in axons towards the posterior of the trunk. Secondary motor neuron (2°MN) axon length as well as branching increased after treatment either with NCI-65828 or terrein (Supplementary Fig. S2).

### Effects on vasculature on treatment with NCI-65828 or terrein prior to MN specification

**Figure 6:**
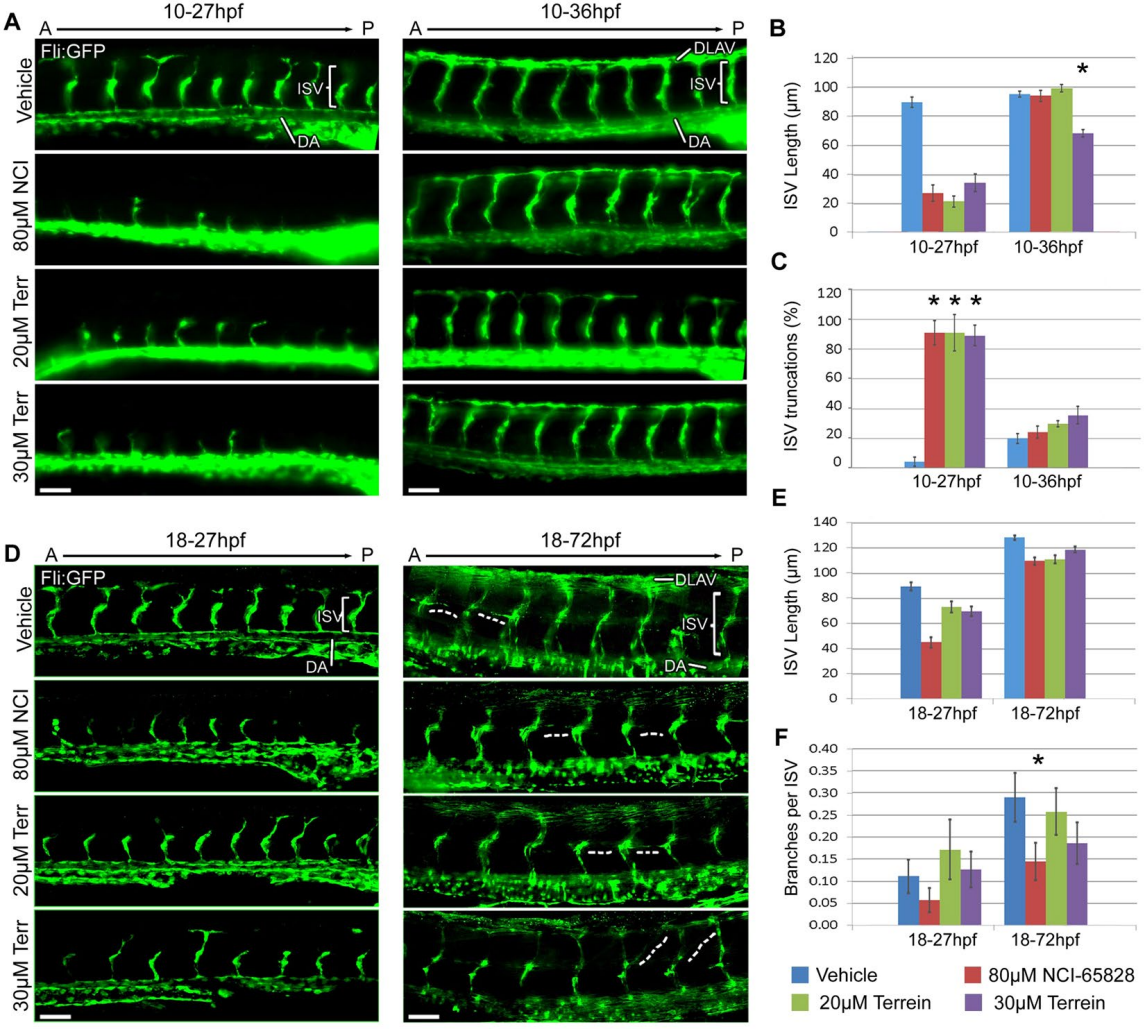
Vascular defects in zebrafish embryos treated with NCI-65828 and terrein. Immunostaining for GFP in Fli:GFP zebrafish treated with terrein or NCI-65828 from 10hpf (**A**) shows retarded inter-somitic vessel formation (ISV) at 27 hpf (**B**) which then recover by 36hpf after NCI-65828 and 20 µM terrein where they span somites from the dorsal aorta (DA) to the dorsal longitudinal anastomotic vessel (DLAV) but not with 30 µM terrein which remain significantly shorter. At 27 hpf over 80% of ISV have not progressed past the midline (**C**). Immunostaining for GFP in Fli:GFP zebrafish treated with terrein or NCI-65828 from 18 hpf (**D**) show similar but less severe retardation of ISV growth at 27 hpf and no difference in ISV length at 72 hpf (**E**). Treatment with NCI-65828 and 30 µM terrein results in less branching of the ISV (**F**) and 30 µM terrein in particular appears to cause aberrant orientations in the branches (**D**, 72 hpf dotted line). Scale bars 50 µm. Error bars SEM. *P < 0.05. Quantification made of trunk vessels above the yolk sac extension from 10 embryos in two replicate experiments.

The dorsal aorta in both inhibitor treated and untreated embryos appeared to have no obvious defects. Inter-somitic vessels (ISV) were seen sprouting in all cases but were more developed in untreated embryos (Fig. 6A). Many ISVs were found to be sprouting laterally at the ventral side in untreated embryos, prior to formation of the dorsolateral anastomotic vessel (DLAV). This was not observed in any of the inhibitor treated embryos where ISV sprouts barely appear to develop past the midline. By 36 hpf, the DLAV was fully developed in untreated embryos whereas in treated embryos, although the ISVs were positioned correctly and reached the dorsal side, the DLAV did not appear as thick or complete.

Growth of ISVs was severely retarded by NCI-65828 as well as both concentrations of terrein at 27 h. Embryos incubated in NCI-65828, 20 µM and 30 µM terrein for 27 h had substantially shorter ISVs (Fig. 6B) but by 36 h the vessels in NCI-65828 had grown to lengths comparable to those of the control. In contrast, ISVs in embryos incubated in 30 µM terrein did not attain the lengths seen in the untreated embryos and vessels stopped around the midline and did not connect with the dorsal vasculature both at 27 and 36 hpf. Besides growth and elongation of ISVs, we also analysed the effects on branching of the blood vessels. At 27 hpf, branching in the ISVs was rare in control embryos but appears slightly reduced in NCI-65828 treated embryos but surprisingly increased in 20 µM terrein alone (Fig. 6C).

### Effects on vasculature on treatment with NCI-65828 or terrein after MN specification

When compared to embryos treated with inhibitors from 10 hpf, the ISVs in embryos from 18hpf appeared to be more developed (Fig. 6D). They were longer and thicker, extended past the midline and sprouted laterally towards the dorsal side. Inter-somitic vessels were shorter in embryos incubated in NCI-65828 or 30 µM terrein from 18.5 hpf to 27 hpf when compared to control. These effects were most striking in NCI-65828 treated embryos. However, in embryos treated (from 18 hpf to 72 hpf) with inhibitors, the ISVs were only marginally shorter suggesting that the shorter ISVs seen earlier are due to growth retardation in inhibitor treated embryos which eventually catch up. ISV truncations were rare in all 72 hpf embryos (Fig. 6E). By 72 hpf, the DLAV appeared well developed in both treated and untreated embryos. At 72 hpf, small vessels could be seen branching from the midline of the embryos. In control embryos these appeared typically perpendicular to the ISVs, however, many were found at incorrect angles after treatment with 30 µM terrein (Fig. 6D; dotted lines, E,F).

At 72 hpf, embryos incubated in the inhibitors from 18.5 hpf developed a dorsal curvature. While untreated fish remained straight and of consistent length, NCI-65828 treated embryos developed mild dorsal curvature. Incubation of embryos in 20 or 30 µM terrein caused a much more noticeable dorsal curvature which was severe in the higher concentration of 30 µM terrein. At the higher terrein concentration, a few embryos presented with shortened tails.

### Other phenotypes in inhibitor-treated embryos

Swimming behaviour was abnormal in treated embryos and also more frequently observed than in controls. Embryos appeared to move using only pectoral fins, no sinusoidal movements using lateral muscles were seen (Supplementary Video S1). The fish appeared to be paralysed, as they were unable to straighten out during any movement and were unable to right themselves. These effects were seen embryos exposed to both inhibitors but were most severe in 30 µM terrein. Hearts beat normally in all cases and blood was observed to be circulating through dorsal and ventral vessels.

### Muscle development is not affected in embryos treated with NCI-65828 or terrein

To determine whether the movement defects we see in the inhibitor treated fish are due effects on the muscles, we examined the patterning of the myotomes. Morphologically there appeared to be no defects in the myotome which was further confirmed by staining with the muscle marker mAb F59^41,42^ (Supplementary Fig. S3**)**.

## Discussion

We have used the well characterised programme of motor neuron development in the zebrafish embryo to demonstrate that the RNase activity of the ANG homolog Rnasels is required for normal developmental progression. By blocking both the RNase activity of the Rnasels using the specific inhibitor NCI-65828 and the secretion of the RNasels by terrein, we show growth retardation and impaired pathfinding in the primary motor neurons of the spine, as well as increased branching in secondary motor neurons. We have previously identified a role for ANG and its mouse homolog in neurite outgrowth *in vitro*^11,14,24^ and here we show this for the first time *in vivo*. Zebrafish have also been used to study the role of other genes implicated in ALS, such as *SOD1* and *C9orf72*^43,44^. The defects we observed shed light on the effects of ALS-associated human ANG variants with active site mutations resulting in grossly impaired or loss of RNase activity (e.g. K40I, K17I) or those with signal peptide mutations predicted to result in impaired secretion (P-4S).

The effects of NCI-65828 and terrein on axon development are similar to the effects of NCI-65828 seen previously in mouse motor neurons differentiated from stem cells in culture^11,14^. Similar effects are seen in perturbation studies of other genes such as survival motor neuron (SMN) and *unplugged* mutants^45–47^ which shows motor neuron truncation and arborisation to a greater extent than that induced by NCI-65828 treatment. *Unplugged* functions as part of the signalling system, guiding motor neuron axons along their stereotypical pathways, and it is possible that Rnasels perform a similar role between motor neurons and their surroundings. This is suggested by the findings of Wei *et al*.^16^, that hANG interacts with molecules such as β-actin and α-actinin. They also showed that hANG is present at the leading edge of migrating cells and cultured mammalian cells failed to migrate properly in the absence of hANG. We have also observed that hANG associates with GAP-43 in the early stages of neuronal differentiation from embryonal carcinoma cells^11^.

The results presented here also suggest that Rnasels have physiological roles in angiogenesis *in vivo* like human ANG. NCI-65828 has been shown to inhibit angiogenesis in HUVEC tube forming assays^9^. Thus ANG like Rnasels function *in vivo* like VEGF in both vascular and neuronal development^48^. Inhibition of Rnasels by NCI-65828 and terrein shown here cause vascular aberrations primarily in the trunk vasculature of embryos which may or may not become more pronounced in adulthood. Rnasels regulate rRNA production and kinase phosphorylation, suggesting a role – even if indirect – in signalling processes^28,49^. Evidence for a direct role comes from characterisation by Chamoux *et al*.^50^, of ANG receptors on capillaries in the bovine brain while signalling pathways have been shown by Gho and Chae^51^ followed by Weidlocha^52^.

Although we cannot exclude the possibility that NCI-65828 acts on other molecules, three lines of evidence, taken together, point to ANG as the most likely target. Kao *et al*.^9^ found that (i) An analogue of NCI-65828 which was significantly lower effect on the enzymatic activity of ANG did not have a similar effect on neovascularization. Minor changes in ligand structure markedly reduced potency, suggesting that the inhibition of ANG activity was through an active-site rather than nonspecific binding which was supported by observations from a computationally generated model of the ANG.65828 complex (ii) Tumours from mice treated with NCI-65828 had fewer blood vessels in the interior of the tumour as compared control groups. This has also been reported for two antagonists that were demonstrably ANG specific (mAb and antisense)^53,54^ (iii) Data reported in the NCI web site (http://dtp.nci.nih.gov/docs/cancer/searches/cancer_open_compounds.html) show that NCI-65828, at concentrations up to 100 μM, did not inhibit the growth in culture of PC-3, HT-29, or any of 57 other human tumour cell lines tested. These data suggest that the effects of NCI-65828 are most likely through its Ang inhibitory activity.

The inhibition of Rnasel activity by NCI-65828 and the inhibition of secretion by terrein occurs ubiquitously throughout the developing embryo, hence it is difficult to conclude whether the defects observed in the developing nervous system are primary or secondary to those seen in the developing vascular system. Both phenotypes are unlikely to be due to general developmental deficits since we did not observe segmental defects in adaxial muscle patterning, defects in anterior/posterior vascular tissues such as the heart, dorsal aorta or cardinal vein, or the positioning of the spinal cord motor neuron nuclei.

Our previous findings from a detailed three-dimensional crystal structure analysis clearly suggest that Rnasel-1 is most similar to hANG (human angiogenin, with a root mean square deviation of 1.6 Å over equivalent 112 C^α^ atoms), including the unique feature such as the active site in obstructed by the C-terminal segment of the molecule (as observed in angiogenin)^27^. The inhibition of Rnasel1 by NCI-65828 is comparable to that observed for hANG. These findings combined with the observations on the spatiotemporal expression of the Rnasels suggest that the effects on the motor neurons and the vascular system are likely to be mediated by Rnasel-1 as it is seen to be expressed at sufficiently high levels in the eye, heart, brain and spine, as with its mouse and human orthologs^11^.

Our detailed study on Rnasel expression and inhibitor studies suggest that hANG-like Rnasels play important roles in axonal pathfinding as well as in angiogenesis in zebrafish. Using an *in vivo* model we demonstrate that the RNase activity and secretion of angiogenins are essential for their function in the development of the nervous system providing insights into the effects seen in ALS patients with mutations in the signal sequence as well as the active site.

## Methods

### RNase assays and inhibition analysis of zebrafish RNases

All RNases (except RNase A- from Sigma-Aldrich) were prepared in [Met^−1^]-form as reported^55^ and authenticated using mass spectrometry. Compound NCI-65828 (Fig. 1D) was the kind gift of Dr Robert Shapiro; NCI-65828 (Tyger Scientific as reported)^9^. The fluorogenic substrate 6-FAM-mAmArCmAmA-Dabcyl was synthesized by Integrated DNA Technologies (Coralville, IA).

Concentrations of protein and 6-FAM-mAmArCmAmA-Dabcyl solutions were determined spectrophotometrically. For the proteins, extinction coefficients calculated by the method of^56^ were used. These were: for RNase A, ε_280_ = 9440 M^−1^ cm^−1^; for Rnasel-1a and -3e, ε_280_ = 13325 M^−1^ cm^−1^; for hAng, ε_280_ = 11835 M^−1^ cm^−1^. For 6-FAM-mAmArCmAmA0Dabcyl, an extinction coefficient calculated by the manufacturer’s OligoAnalyzer 3.1 program was used: ε_260_ = 91043 M^−1^ cm^−1^. Master stock of NCI-65828 was prepared at concentrations of 10 mM and 20 mM, respectively, by the dissolution of weighed solid in DMSO. All working inhibitor solutions were derived from these stocks.

### Kinetic analyses

A modification of earlier fluorimetric methods^9,57^ was employed. Briefly, assays were conducted at 37 °C in a Perkin-Elmer LS-50B fluorimeter using stoppered semi-micro quartz cuvettes (Starna Scientific, Essex, UK). The fluorescence increase accompanying substrate cleavage was monitored using λ_ex_ = 495 nm (slit width fixed at 5 nm) and λ_em_ = 520 nm (slit width varied depending on quenching). Assay mixtures (1 ml) contained 0.02 M HEPES·NaOH, 0.1 M NaCl (pH 7.0), 0.002% (w/v) Tween-20 and 100 nM 6-FAM–mAmArCmAmA–Dabcyl. To each assay, either 20 µl DMSO or 20 µl inhibitor solution (solvent = DMSO) were added. Fluorescence was recorded during a 10-min equilibration period, after which the reaction was initiated by addition of 10 µl of enzyme stock to give a final concentration of either 195 nM Rnasel-1a, 77 nM Rnasel-3e or 370 nM hANG. The progress curve was recorded for 20–25 min, after which time 5 µl of 20 µM RNase A was added to take the reaction to completion. Recording continued until the reading stabilized (typically after 10 min).

Initial trials revealed that inclusion of 0.002% (w/v) Tween-20 as a surface-blocking agent increased initial rates by 10–20% and improved the linearity of the initial phase of the progress curve when compared with equivalent assays that included 10 µg/ml BSA^9^ instead. Initial rate (*v*), initial fluorescence (*F*_0_) and maximal fluorescence (*F*_max_) were obtained using Grafit 5 (Erithacus Software, Surrey, UK). For *v* and *F*_0_ estimation, a straight line was fitted to the data from the first 10 min of the progress curve, corresponding to ≤ 2% substrate cleavage. When progress curves showed a transient, data from the 10 min immediately following the transient were used. It was expected that [S] <<*K*_m_^58,59^, hence *k*_cat_/*K*_m_ was calculated as *v*/((*F*_max_ − *F*_0_)[E]), this being equal to *v*_0_/([E][S]) in molar terms^60,61^. IC_50_ values were obtained by fitting dose-response data to a sigmoid curve of standard slope:

$${({k}_{{\rm{cat}}}/{K}_{{\rm{m}}})}_{{\rm{i}}}={({k}_{{\rm{cat}}}/{K}_{{\rm{m}}})}_{{\rm{0}}}/(1+{10}^{(\mathrm{log}([{\rm{I}}])\mbox{--}{\rm{X}})})$$

or to one of variable slope:

$${({k}_{{\rm{cat}}}/{K}_{{\rm{m}}})}_{{\rm{i}}}={({k}_{{\rm{cat}}}/{K}_{{\rm{m}}})}_{0}/(1+{10}^{((\mathrm{log}([{\rm{I}}])\mbox{--}{\rm{X}})\ast h)})$$

where (*k*_cat_/*K*_m_)_i_ is the value of *k*_cat_/*K*_m_ in the presence of inhibitor, (*k*_cat_/*K*_m_)_0_ is a constant equal to the value of *k*_cat_/*K*_m_ in the absence of inhibitor, [I] is the inhibitor concentration, X is a term equal to log(IC_50_), and *h* is the Hill slope.

### Terrein treatment and Western blot of SH-SY5Y expressing HA tagged mAng1

Near confluent SH-SY5Y mAng1HA cells were switched from serum-containing growth medium to DMEM:F12 with 1% NEAA and 5 mM Glutamax (Invitrogen) with or without 30 μM terrein (Sigma). After 24 or 48 h cells were washed twice with PBS then lysed in reducing SDS-PAGE loading buffer [2% SDS (Sigma), 10% Glycerol (BDH), 60 mM Tris (Sigma), 100 mM DTT (Sigma)] and protein concentration determined using Biorad Protein Assay. 50 µg of each sample was denatured by boiling for 5 min then run on a 10% Tris-Tricine gel alongside Fermentas PageRuler Unstained Low Range Protein Ladder. Wet transfer was performed at 30 V for 1 h to PVDF membrane (Pierce), which was then blocked in 5%Marvel 0.1% Tween20 (Sigma) in PBS for 1 h RT then incubated with mouse anti-HA (Covance) 1:5000 overnight at 4 °C. The membrane was then washed 4 × 5 min with PBST and incubated with HRP conjugated anti-mouse (Sigma) 1:5000 for 2 h at RT. After another 4 × 5 min wash with PBST the membrane was incubated with ECL reagents and exposed to Amersham Hyperfilm MP. GAPDH was used as loading control, HRP conjugated anti-GAPDH (Abcam) was used as above at 1:5000.

### Treatment of SH-SY5Y and SH-SY5Y mAng1HA cell lines with terrein

SH-SY5Y and SH-SY5Y expressing HA-tagged mAng1 (SH-SY5Y mAng1HA) were maintained in DMEM:F12 (Invitrogen) supplemented with 10% FBS (Biosera), 1% Non-essential amino acids (Invitrogen) and 1% Glutamax (Invitrogen)) on 10 cm dishes (BD Falcon). SH-SY5Y cell lines were seeded at a density of 10^5^ cells/cm^2^ on acid-washed coverslips (SLS) in 24-well plates (BD Falcon) in complete medium. Medium was exchanged after 24 h with complete medium containing 0, 15, 20 or 30 µM terrein. Cells were cultured in the presence of terrein for the indicated period then fixed with 4% PFA on ice for 15 min. After fixing cells were washed twice with PBS and dehydrated to 70% ethanol through 30% and 50% in ten minute incubations each. For western blotting cells were lysed in a reducing and denaturing buffer containing 2% SDS (Sigma), 10% Glycerol (BDH), 60 mM Tris (Sigma) and 100 mM DTT (Sigma).

### Immunocytochemistry

Fixed cells were rehydrated to PBS and washed twice for ten minutes before blocking for 1 h at room temperature in PBS with 0.1% gelatin, 0.5% FBS and 0.1% Triton-X100, and incubated with primary antibodies overnight in the same buffer. After washing four times with PBST (PBS with 0.1% Triton-X100) for ten minutes each cells were incubation with secondary antibodies for two hours again in blocking buffer. After a further four PBST washes, samples were mounted with Mowiol. Z-stacked images were acquired using a Leica DM5500B microscope, DFC 360FX camera and LAS software and deconvoluted. See Table 1 for antibody information.

**Table 1:** Antibodies used in this study and dilutions.

Antibody	Isoform	Dilution	Supplier/Cat.#
ZnP1	Mouse,IgG2a	1:50	ZFIN, ZDB-ATB-081002-25
SV2	Mouse,IgG1	1:5	DSHB
F59 (MHC)	Mouse, IgG1	1:5	DSHB
40-2D6 (Isl1/2)	Mouse, IgG1	1:5	DSHB
Rabbit α GFP	Rabbit	1:500	Invitrogen, A-11122
PDI	Rabbit	1:200	Cell Signalling Technologies, #3501
TGN46	Rabbit	1:500	Abcam, Ab16052
LAMP1	Rabbit	1:200	Abcam, Ab51157
Goat α mouse, Alexa fluor 488	Goat, IgG2a	1:2000	Invitrogen, A-21131
Goat α mouse Alexa fluor 594	Goat, IgG (H + L)	1:2000	Invitrogen, A-11004
Goat α rabbit Alexa fluor 488	Goat, IgG (H + L)	1:2000	Invitrogen, A-11008

### Quantification of cleaved Caspase 3

Counts of total DAPI positive and cleaved caspase 3 positive cells were made in five randomly selected fields on each coverslip from each condition from two independent experiments. Each field contained a mean of 600 cells. Data presented is the mean of the percentage of total cells positive for cleaved caspase 3.

### Zebrafish breeding

ABWT Zebrafish^62^ were kept in tanks (Aquatic Habitats system, S66U-2 model) at 28 °C containing water of pH 7.5–8.0 and were fed brine shrimp. Breeding was conducted using two pairs of four-month-old fish per breeding tank in a light-dark cycled 28 °C incubation chamber. Light-dark cycle consisted of 10 hours dark and 14 hours light. Fish were acclimated overnight and permitted to breed at first light. When breeding was successful eggs were collected at 1 hpf and kept in E3 saline solution containing methylene blue during development. *Tg*(*fli1a:EGFP*) embryos were provided by Dr Makoto Furutani Seiki. All animals were handled in accordance with the relevant national guidelines. The zebrafish used in these experiments are housed in a facility certified by the UK Home Office, and the work was approved by the University of Bath Animal Welfare and Ethical Review Body All experiments were performed in accordance with the UK (Animal Procedures) Act 1986.

### RNA isolation and quantitative RT-PCR for Zebrafish RNase transcripts

RNA was prepared from organs isolated from adult or embryos of the indicated stages by Trizol following manufacturer’s instructions. Organs from four fish were pooled after individual homogenisation using a Dounce homogeniser. Pooled clutches with a minimum of 100 timed staged embryos were processed in the same manner. Samples were DNased using Turbo DNase-free (Ambion) and reverse transcribed by M-MuLV H-minus reverse transcriptase for one hour at 42 °C and inactivated at 70 °C for five minutes (RevertAid™ H Minus Reverse Transcriptase kit, Fermentas).

Mastermix comprising iQ SYBR Green supermix (BioRad), water and cDNA were made for quantitative PCR. Appropriate primers were added and divided into three replicates for each gene on PCR plates (Thermo). Final reactions were 20 µl in volume with 0.1 µM primers and 10 ng cDNA (see Table 2 for primer sequences). Reactions were carried out in a BioRad iQ5 cycler. Dynamic well factors were collected for 2 min 30 sec, then forty cycles at 60 °C and 95 °C for 20 s each followed by a melt curve. Expression levels were determined relative to 18 s rRNA^63^ from baseline subtracted curves and corrected using primer efficiencies determined previously from serial dilutions of PCR product. Each was performed on cDNA from two individual pooled adult organ or embryonic preparations.

**Table 2:** Primers used in qRT-PCR experiments.

Target gene	Forward (5′-3′)	Reverse (5′-3′)	Reference
Rnasel-1	ACCAGCATGTGGGACCTGAT	CCTGTGGACTTCCTGCTCGG	FB58B02.Y1, Primer BLAST
Rnasel-2	TGGTCGACACGAACCTGACTAA	GGCGTCTCTCCCCACTTTTTA	Quarto^26^
Rnasel-3	CACTTACGGTCAACCAGCAGAAA	TGTCTGCTCAGTTATGCCTCCAT	Quarto^26^
18 srRNA	TCGCTAGTTGGCATCGTTTATG	CGGAGGTTCGAAGACGATCA	McCurley^63^

### Treatment of zebrafish embryos with NCI-65828 and terrein

Embryos were collected in E3 at 28 °C then sorted and synchronised at shield stage (6hpf). One hour prior to drug addition, embryos were dechorionated using Pronase (Sigma) and washed extensively in E3 without methylene blue. Methylene blue was omitted from all subsequent E3 during drug treatment due to a reaction with NCI-65828 resulting in precipitation. Up to 50 embryos were transferred to 1.5% agarose coated 6-well plates (BD Falcon) in 2 ml of E3. Drugs were prepared as 100x stocks in DMSO; 8 mM NCI-65828 and either 2 mM or 3 mM terrein. At either 10 or 18hpf drugs were added to a final concentration of 80 µM NCI-65828, 20 µM terrein and 30 µM terrein with 0.1% DMSO used as control. 0.2 mM 1-phenyl 2-thiourea (PTU) was added at 24hpf. Observations were made using a Leica MZ8 stereoscope with images acquired with LAS AF (Leica) and videos recorded through VLC (videolan.org) through a Leica DFC490 camera. Embryos were washed twice with E3 before fixation in 4% paraformaldehyde (PFA, Sigma) pH7.4 overnight at 4 °C. After fixation embryos were washed twice with PBS and dehydrated through 30%, 50%, 70% to 80% methanol, each for 1 h.

### Wholemount immunohistochemistry

Ten to twenty embryos were rehydrated to PBS and washed four times for 30 minutes with 1 ml incubation buffer (IB; 1% bovine serum albumen (Sigma), 0.5% Triton X100 (Sigma) and 1% DMSO in PBS). After a final 30 minute wash in IB with 1% goat serum (Sigma) embryos were incubated overnight at 4 °C in the same buffer with rocking using primary antibodies at the dilutions indicated in Table 2. Following four washes with 1 ml IB embryos were again incubated overnight at 4 °C with rocking with secondary antibodies diluted in IB with 1% goat serum. After one final wash with IB, three further washes in PBS were performed for 30 min each.

### Mounting and imaging of fluorescently-labelled embryos

Embryos were de-yolked using pins and briefly washed with PBS to remove debris then cleared through 25%, 50% to 70% glycerol (VWR) in PBS before mounting and imaging. Heads were cut from the trunk just posterior to the otic vesicles before mounting in glycerol under a coverslip (Thermo, 18 × 18 mm, #1.5) supported by vaseline. Trunks were mounted right-side down while heads were mounted ventral-side down.

Embryos were viewed using a Leica DM5500B microscope fitted with a Leica CTR5500 lightbox and DFC360FX camera. Images were acquired using Leica Application Software (LAS) version 2.1.2, Z-stacks of images were deconvoluted in the same software.

### Quantification of zebrafish trunk motor neurons and intersomitic vessels

Measurements were taken from seven trunk motor neurons starting from immediately posterior to where the yolk sac joins the yolk proper and identified by Znp1 staining. Neurite lengths and branching were measured by tracing through 3D volumes in Z-stacks using the Fiji distribution of imageJ (fiji.sc/Fiji) and the plug-in Simple Neurite Tracer (fiji.sc/Simple_Neurite_Tracer). The same process was used to quantify inter-somitic vessel (ISV) length and branching, identified by Fli:GFP expression. Truncations were scored manually where ISV failed to reach the dorsal longitudinal anastomotic vessel and observations were made of sprouting at the midline. Motor neuron nuclei identified by Islet staining were counted in the same region and analysed as three equal-sized bins corresponding to the anterior, middle and posterior area. To ensure any defects seen were not due to underlying body-plan defects, observations were made of myosin heavy chain staining (F59) in the same region, none were found.

### Statistics

All statistical tests were performed in SPSS 22 (IBM). Neurite and vascular quantification was performed on at least ten embryos from each time-point from two experiments were used. Measurements from neurons in equivalent positions posterior to the yolk-yolk sac boundary were pooled between fish in the same group and compared between treatments. Measurements were made from both left and right-side neurons and vasculature and although the same defects were identified consistently different lengths were obtained for equivalent left and right side neurons in fish within the same group (control and treated). These may have been an artefact due to imaging through the embryo for right-side neurons. As these were present in the control also, only the left side data has been presented here. Normality was tested using the D’Agostino-Pearson normality test prior to ANOVA testing with Tukey’s post-hoc.

### Figure compositions and Movie

Graphs were created in either Excel (Microsoft) or SPSS 22. Images were exported from LAS AF. Figures were composed in Photoshop CS3 (Adobe). Video figure created in Windows Movie Maker (Microsoft).

## Supplementary information

Supplementary Material

Supplementary Video

## References

[1] Fett JW (1985). Isolation and characterization of angiogenin, an angiogenic protein from human carcinoma cells. Biochemistry.

[2] Strydom DJ (1985). Amino acid sequence of human tumor derived angiogenin. Biochemistry.

[3] Shapiro R, Riordan JF, Vallee BL (1986). Characteristic ribonucleolytic activity of human angiogenin. Biochemistry.

[4] Shapiro R, Strydom DJ, Olson KA, Vallee BL (1987). Isolation of angiogenin from normal human plasma. Biochemistry.

[5] Kurachi K, Davie EW, Strydom DJ, Riordan JF, Vallee BL (1985). Sequence of the cDNA and gene for angiogenin, a human angiogenesis factor. Biochemistry.

[6] Moroianu J, Riordan JF (1994). Nuclear translocation of angiogenic proteins in endothelial cells: an essential step in angiogenesis. Biochemistry.

[7] Moroianu J, Riordan JF (1994). Identification of the nucleolar targeting signal of human angiogenin. Biochem. Biophys. Res. Commun..

[8] Moroianu J, Riordan JF (1994). Nuclear translocation of angiogenin in proliferating endothelial cells is essential to its angiogenic activity. Proc. Natl. Acad. Sci. USA.

[9] Kao RYT (2002). A small-molecule inhibitor of the ribonucleolytic activity of human angiogenin that possesses antitumor activity. Proc. Natl. Acad. Sci. USA.

[10] Arakawa M, Someno T, Kawada M, Ikeda D (2008). A new terrein glucoside, a novel inhibitor of angiogenin secretion in tumor angiogenesis. J. Antibiot. (Tokyo).

[11] Subramanian V, Feng Y (2007). A new role for angiogenin in neurite growth and pathfinding: implications for amyotrophic lateral sclerosis. Hum. Mol. Genet..

[12] Sebastià J (2009). Angiogenin protects motoneurons against hypoxic injury. Cell Death Differ..

[13] Kieran D (2008). Control of motoneuron survival by angiogenin. J. Neurosci..

[14] Subramanian V, Crabtree B, Acharya KR (2008). Human angiogenin is a neuroprotective factor and amyotrophic lateral sclerosis associated angiogenin variants affect neurite extension/pathfinding and survival of motor neurons. Hum. Mol. Genet..

[15] Wu D (2007). Angiogenin loss-of-function mutations in amyotrophic lateral sclerosis. Ann. Neurol..

[16] Wei S, Gao X, Du J, Su J, Xu Z (2011). Angiogenin enhances cell migration by regulating stress fiber assembly and focal adhesion dynamics. PloS One.

[17] Adams SA, Subramanian V (1999). The angiogenins: an emerging family of ribonuclease related proteins with diverse cellular functions. Angiogenesis.

[18] Greenway MJ (2004). A novel candidate region for ALS on chromosome 14q11.2. Neurology.

[19] Gellera C (2008). Identification of new ANG gene mutations in a large cohort of Italian patients with amyotrophic lateral sclerosis. Neurogenetics.

[20] Fernández-Santiago R (2009). Identification of novel Angiogenin (ANG) gene missense variants in German patients with amyotrophic lateral sclerosis. J. Neurol..

[21] van Es MA (2009). A Case of Als-Ftd in a Large Fals Pedigree with a K17i Ang Mutation. Neurology.

[22] van Es MA (2011). Angiogenin variants in Parkinson disease and amyotrophic lateral sclerosis. Ann. Neurol..

[23] Greenway MJ (2006). ANG mutations segregate with familial and ‘sporadic’ amyotrophic lateral sclerosis. Nat. Genet..

[24] Thiyagarajan N, Ferguson R, Subramanian V, Acharya KR (2012). Structural and molecular insights into the mechanism of action of human angiogenin-ALS variants in neurons. Nat. Commun..

[25] Pizzo E (2006). Ribonucleases and angiogenins from fish. J. Biol. Chem..

[26] Quarto N, Pizzo E, D’Alessio G (2008). Temporal and spatial expression of RNases from zebrafish (*Danio rerio*). Gene.

[27] Kazakou K, Holloway DE, Prior SH, Subramanian V, Acharya KR (2008). Ribonuclease A homologues of the zebrafish: polymorphism, crystal structures of two representatives and their evolutionary implications. J. Mol. Biol..

[28] Monti DM (2009). Characterization of the angiogenic activity of zebrafish ribonucleases. FEBS J..

[29] Cho S, Zhang J (2007). Zebrafish ribonucleases are bactericidal: implications for the origin of the vertebrate RNase A superfamily. Mol. Biol. Evol..

[30] Lawson ND, Weinstein BM (2002). *In vivo* imaging of embryonic vascular development using transgenic zebrafish. Dev. Biol..

[31] Bandmann O, Burton EA (2010). Genetic zebrafish models of neurodegenerative diseases. Neurobiol. Dis..

[32] Patten SA (2014). Fishing for causes and cures of motor neuron disorders. Dis. Model. Mech..

[33] Li S, Yu W, Hu G-F (2012). Angiogenin inhibits nuclear translocation of apoptosis inducing factor in a Bcl-2-dependent manner. J. Cell. Physiol..

[34] Kimmel CB, Ballard WW, Kimmel SR, Ullmann B, Schilling TF (1995). Stages of embryonic development of the zebrafish. Dev. Dyn..

[35] Beattie CE (2000). Control of motor axon guidance in the zebrafish embryo. Brain Res. Bull..

[36] Fouquet B, Weinstein BM, Serluca FC, Fishman MC (1997). Vessel patterning in the embryo of the zebrafish: guidance by notochord. Dev. Biol..

[37] Isogai S, Horiguchi M, Weinstein BM (2001). The vascular anatomy of the developing zebrafish: an atlas of embryonic and early larval development. Dev. Biol..

[38] Isogai S, Lawson ND, Torrealday S, Horiguchi M, Weinstein BM (2003). Angiogenic network formation in the developing vertebrate trunk. Dev. Camb. Engl..

[39] Appel B (1995). Motoneuron fate specification revealed by patterned LIM homeobox gene expression in embryonic zebrafish. Dev. Camb. Engl..

[40] Myers PZ, Eisen JS, Westerfield M (1986). Development and axonal outgrowth of identified motoneurons in the zebrafish. J. Neurosci..

[41] Crow MT, Stockdale FE (1986). The developmental program of fast myosin heavy chain expression in avian skeletal muscles. Dev. Biol..

[42] Devoto SH, Melançon E, Eisen JS, Westerfield M (1996). Identification of separate slow and fast muscle precursor cells *in vivo*, prior to somite formation. Dev. Camb. Engl..

[43] Lemmens R (2007). Overexpression of mutant superoxide dismutase 1 causes a motor axonopathy in the zebrafish. Hum. Mol. Genet..

[44] Ciura S (2013). Loss of function of C9orf72 causes motor deficits in a zebrafish model of amyotrophic lateral sclerosis. Ann. Neurol..

[45] McWhorter ML, Monani UR, Burghes AHM, Beattie CE (2003). Knockdown of the survival motor neuron (Smn) protein in zebrafish causes defects in motor axon outgrowth and pathfinding. J. Cell Biol..

[46] Granato M (1996). Genes controlling and mediating locomotion behavior of the zebrafish embryo and larva. Dev. Camb. Engl..

[47] Zhang J, Granato M (2000). The zebrafish unplugged gene controls motor axon pathway selection. Dev. Camb. Engl..

[48] Suchting S, Bicknell R, Eichmann A (2006). Neuronal clues to vascular guidance. Exp. Cell Res..

[49] Xu Z, Tsuji T, Riordan JF, Hu G (2003). Identification and characterization of an angiogenin-binding DNA sequence that stimulates luciferase reporter gene expression. Biochemistry.

[50] Chamoux M (1991). Characterization of angiogenin receptors on bovine brain capillary endothelial cells. Biochem. Biophys. Res. Commun..

[51] Gho YS, Chae CB (1997). Anti-angiogenin activity of the peptides complementary to the receptor-binding site of angiogenin. J. Biol. Chem..

[52] Wiedłocha A (1999). Following angiogenin during angiogenesis: a journey from the cell surface to the nucleolus. Arch. Immunol. Ther. Exp. (Warsz.).

[53] Olson KA, Byers HR, Key ME, Fett JW (2001). Prevention of Human Prostate Tumor Metastasis in Athymic Mice by Antisense Targeting of Human Angiogenin. Clin Cancer Res.

[54] Olson, K. A., Byers, H. R., Key, M. E. & Fett, J. W. Inhibition of prostate carcinoma establishment and metastatic growth in mice by an antiangiogenin monoclonal antibody. *Int J Cancer*. **98**, 923–929 (2002). 10.1002/ijc.10282 11948474

[55] Holloway DE, Hares MC, Shapiro R, Subramanian V, Acharya KR (2001). High-level expression of three members of the murine angiogenin family in *Escherichia coli* and purification of the recombinant proteins. Protein Expr. Purif..

[56] Pace CN, Vajdos F, Fee L, Grimsley G, Gray T (1995). How to measure and predict the molar absorption coefficient of a protein. Protein Sci. Publ. Protein Soc..

[57] Kelemen BR (1999). Hypersensitive substrate for ribonucleases. Nucleic Acids Res..

[58] Russo N, Shapiro R, Acharya KR, Riordan JF, Vallee BL (1994). Role of glutamine-117 in the ribonucleolytic activity of human angiogenin. Proc. Natl. Acad. Sci. USA.

[59] Leland PA, Staniszewski KE, Park C, Kelemen BR, Raines RT (2002). The ribonucleolytic activity of angiogenin. Biochemistry.

[60] Fersht, A. *Structure and mechanism in protein science: A guide to enzyme catalysis and protein folding*. (W. H. Freeman, 1999).

[61] Copeland, R. A. *Enzymes: A practical introduction to structure*, *mechanism*, *and data Analysis*. (John Wiley & Sons, 2004).

[62] Westerfield, M. *The zebrafish book*. *A guide for the laboratory use of zebrafish (Danio rerio)*. (The University of Oregon Press, 2003).

[63] McCurley AT, Callard GV (2008). Characterization of housekeeping genes in zebrafish: male-female differences and effects of tissue type, developmental stage and chemical treatment. BMC Mol. Biol..

