# Supplementary material for: The catalytic activity and secretion of zebrafish RNases are essential for their in vivo function in motor neurons and vasculature
Source: Sci Rep. 2019 Feb 1;9:1107. doi: 10.1038/s41598-018-37140-2 (PMC6358602; doi:10.1038/s41598-018-37140-2)
Supplement: Supplementary file 1 — Supplementary Material [file 41598_2018_37140_MOESM1_ESM.docx]

**The catalytic activity and secretion of zebrafish RNases are essential for their *in vivo* function in motor neurons and vasculature**

**Ross Ferguson^1^, Daniel E. Holloway^1^, Anand Chandrasekhar^2^, K. Ravi Acharya^1^ & Vasanta Subramanian^1,^***

^1^Department of Biology and Biochemistry, University of Bath, Bath BA2 7AY, UK

^2^Division of Biological Sciences and Bond Life Sciences Center, University of Missouri, Columbia MO 65211-7310, USA

**Supplementary Information**

Supplementary Figures and Legends


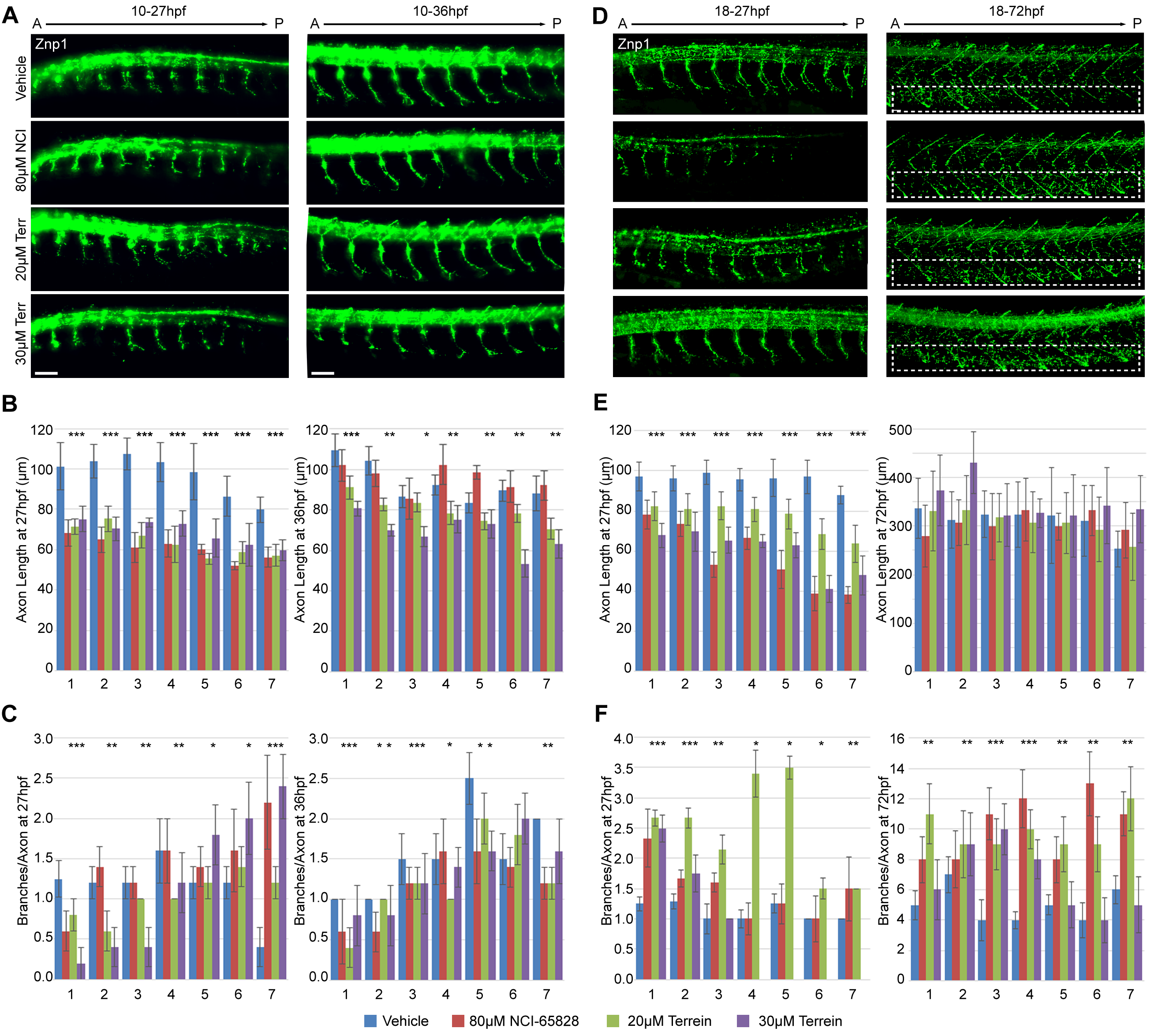


**Supplementary Fig. S1 – Quantification of neuronal defects in terrein treated zebrafish**

(a) Immunostaining for Znp1 in Fli:GFP zebrafish treated with Terrein or NCI65828 from 10hpf and observed at 27 or 36 hpf. (b) Immunostaining for Znp1 in Fli:GFP zebrafish treated with terrein or NCI65828 from 18 hpf and observed 27 or 72 hpf. (b) Quantification of primary motor neuron axon length at the indicated time after treatment from 10hpf. (c) Quantification of primary motor neuron branching at the indicated time after treatment from 10 hpf. (d) Immunostaining for Znp1 in Fli:GFP zebrafish treated with Terrein or NCI-65828 from 18 hpf to 27 or 72 hpf. (e) Quantification of primary motor neuron axon length at the indicated time after treatment from 18 hpf. (f) Quantification of primary motor neuron branching at the indicated time after treatment from 18 hpf. Scale bars 50µm. Error bars SEM. * = P<0.05. Quantification made of seven motor axon projections on the left side (1-7, anterior to posterior) above the yolk sac extension from two replicate experiments of ten embryos each.


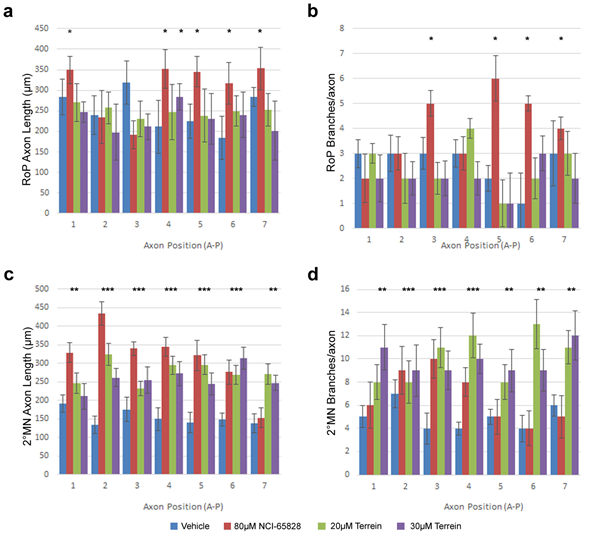


**Supplementary Fig. S2 – Further quantification shows defects in additional neurons at 72hpf**

(a) Quantification of RoP motor neuron axon length at 72 hpf after treatment from 18 hpf in Fli:GFP zebrafish treated with terrein or NCI-65828 from 18 hpf. (b) Quantification of RoP branching at 72 hpf after treatment from 18 hpf. (c) Quantification of secondary motor neuron axon length at 72h after treatment from 18 hpf. (d) Quantification of secondary motor neuron branching at 72h after treatment from 18 hpf. Scale bars 50µm. Error bars SEM. * = P<0.05 Quantification made of seven left side neurons (1-7, anterior to posterior) above the yolk sac extension from 10 embryos in two replicates.


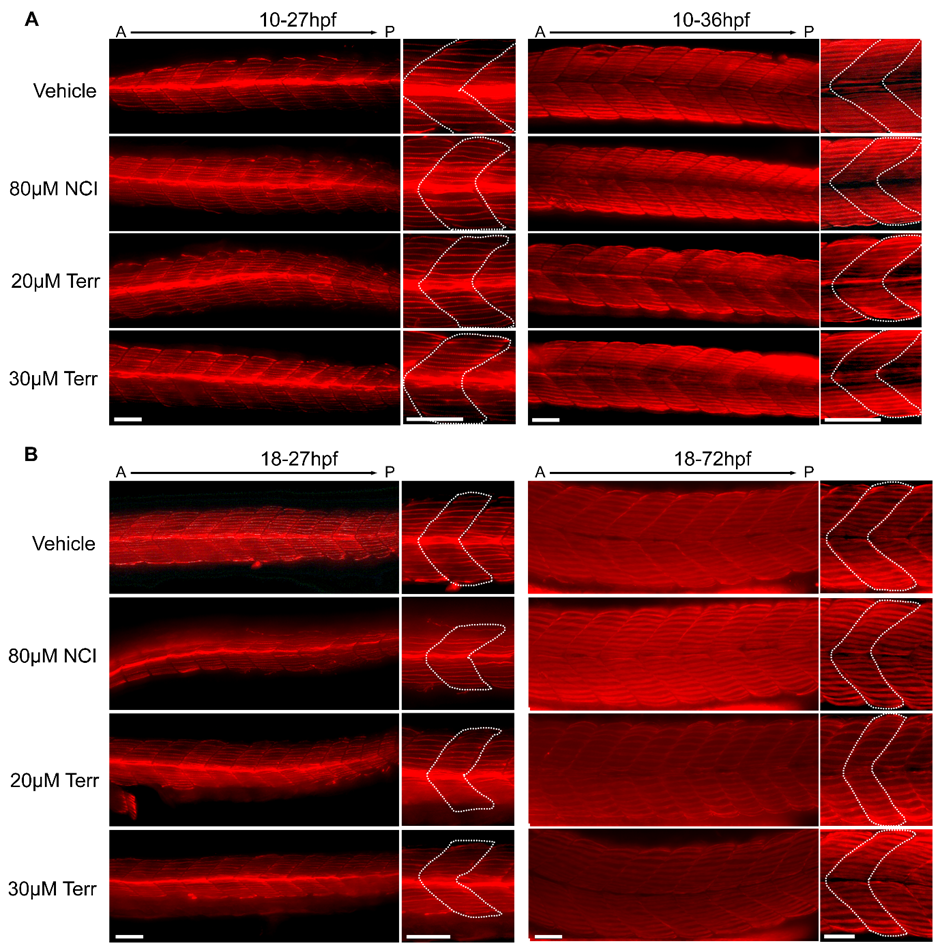


**Supplementary Fig. S3 - Myosin heavy chain immunostaining shows no disruption to underlying body plan after treatment with NCI-65828 or terrein**

Immunostaining for Myosin heavy chain in Fli:GFP zebrafish treated with terrein or NCI65828 from 10hpf to 27 or 36 hpf (a) or 18 hpf to 27 or 72 hpf (b). Insets show enlarged regions of main figure with one unit bounded by a dotted line. Scale bars 50µm.

**Supplementary Video – Zebrafish embryos treated with NCI-65828 or Terrein develop motor defects**

Embryos were treated at 18 hpf, prior to axonal outgrowth but after primary motor neuron specification, with either 80µM NCI-65828, 20µM Terrein or 30uM Terrein. (a) Observations made at 27 hpf show normal development at the gross scale of both treated and untreated embyros. At 72 hpf (b) control fish can be seen occasionally swimming normally, while treated fish have severely arched backs, most sever in 30µM Terrein treated fish. Movement in treated fish appears to be mainly be pectoral fin resulting in a distinctive pinwheel movement due to their rigid arched body. A characteristic red stain can be seen in embryos treated with NCI-65828.
